# DNA Methylation Signature of a Lifestyle-based Resilience Index for Cognitive Health

**DOI:** 10.21203/rs.3.rs-5423573/v1

**Published:** 2024-11-27

**Authors:** Wei Zhang, David Lukacsovich, Juan I. Young, Lissette Gomez, Michael A. Schmidt, Eden R. Martin, Brian W. Kunkle, Xi Chen, Deirdre M. O’Shea, James E. Galvin, Lily Wang

**Affiliations:** 1 Division of Biostatistics, Department of Public Health Sciences, University of Miami, Miller School of Medicine, Miami, FL 33136, USA; 2 Dr. John T Macdonald Foundation Department of Human Genetics, University of Miami, Miller School of Medicine, Miami, FL 33136, USA; 3 John P. Hussman Institute for Human Genomics, University of Miami Miller School of Medicine, Miami, FL 33136, USA; 4 Comprehensive Center for Brain Health, Department of Neurology, University of Miami Miller School of Medicine, Miami, FL 33433, USA; 5 Sylvester Comprehensive Cancer Center, University of Miami, Miller School of Medicine, Miami, FL 33136, USA

**Keywords:** Alzheimer’s disease, cognitive resilience, DNA methylation, lifestyle factors

## Abstract

Cognitive resilience (CR) contributes to the variability in risk for developing and progressing in Alzheimer’s disease (AD) among individuals. Beyond genetics, recent studies highlight the critical role of lifestyle factors in enhancing CR and delaying cognitive decline. DNA methylation (DNAm), an epigenetic mechanism influenced by both genetic and environmental factors, including CR-related lifestyle factors, offers a promising pathway for understanding the biology of CR. We studied DNAm changes associated with the Resilience Index (RI), a composite measure of lifestyle factors, using blood samples from the Healthy Brain Initiative (HBI) cohort. After corrections for multiple comparisons, our analysis identified 19 CpGs and 24 differentially methylated regions significantly associated with the RI, adjusting for covariates age, sex, *APOE ε4*, and immune cell composition. The RI-associated methylation changes are significantly enriched in pathways related to lipid metabolism, synaptic plasticity, and neuroinflammation, and highlight the connection between cardiovascular health and cognitive function. By identifying RI-associated DNAm, our study provided an alternative approach to discovering future targets and treatment strategies for AD, complementary to the traditional approach of identifying disease-associated variants directly. Furthermore, we developed a Methylation-based Resilience Score (MRS) that successfully predicted future cognitive decline in an external dataset from the Alzheimer’s Disease Neuroimaging Initiative (ADNI), even after accounting for age, sex, *APOE ε4*, years of education, baseline diagnosis, and baseline MMSE score. Our findings are particularly relevant for a better understanding of epigenetic architecture underlying cognitive resilience. Importantly, the significant association between baseline MRS and future cognitive decline demonstrated that DNAm could be a predictive marker for AD, laying the foundation for future studies on personalized AD prevention.

## INTRODUCTION

Alzheimer’s disease (AD) is a significant public health problem, affecting millions of people worldwide. A major barrier impeding precision medicine in AD is the substantial variability in risk for developing and progressing in the disease among different individuals. Cognitive resilience (CR) refers to an individual’s adaptability to brain changes due to disease, injury, or normal aging.^1,2^ Higher CR is associated with a lower risk of progression to clinical AD, and a slower rate of cognitive decline.^3^

Several mechanisms have been proposed for CR, including cognitive reserve (greater adaptability of cognitive processes to perform tasks in response to brain aging, pathology, or insult), brain reserve (large neurobiological capital such as a higher number of neurons, white matter connections), and brain maintenance (maintaining brain structure and function) ^4,5^. In addition to genetics^6,7^, recent studies have shown that lifestyle factors, such as engaging in physical activity and following the MIND diet, play important roles in promoting CR and delaying cognitive decline ^8–21^.

Two common approaches have been used to estimate CR: (1) the *proxy approach*, which estimates CR-based activities thought to promote CR, such as education, intellectually engaging occupations, and other activities, or a composite of these activities.^17,18,22–24^; and (2) the *residual approach*, which estimates CR by calculating the difference between observed cognitive performance and expected cognitive performance given a subject’s neuropathology burden^1,2,6,25–27^.

For the proxy approach, we recently developed and validated the Resilience Index (RI), which is composed of measures of six lifestyle factors: cognitive reserve, social, physical and cognitive activity, diet, and mindfulness.^28^ We demonstrated that subjects with higher RI scores had better cognitive, functional, and global outcomes compared to those with lower RI scores, even within the same diagnosis group.

However, to date, the biology underlying CR remains not well understood. While recent studies have explored the biology of cognitive resilience through genetic and protein-based analyses, ^6,29–32^ few have examined the role of epigenetics. DNA methylation (DNAm) is the most widely studied epigenetic mechanism, and is influenced by both genetic and environmental factors, including several CR-related lifestyle factors, such as smoking, diet, and exercise^33^. Moreover, DNAm has been implicated in cognitive decline associated with aging and AD, ^34–36^ suggesting it may serve as a potential marker through which lifestyle factors influence cognition.

In this study, to better understand the biology of CR, we identified DNAm patterns associated with the Resilience Index, by performing a comprehensive analysis of DNAm data generated from blood samples of participants in the Healthy Brain Initiative (HBI) study ^37^. To understand the functional roles of the RI-associated DNAm, we conducted integrative analyses combining DNAm data with blood gene expression data, GWAS summary statistics, and biological pathway databases. Additionally, we developed the Methylation-based Resilience Score (MRS), a methylation-based predictor of the RI, and assessed its potential to predict future cognitive impairment and AD using an external dataset with independent samples. Our findings enhance the understanding of epigenetics underlying lifestyle factors associated with cognitive resilience and suggest that methylation biomarkers could serve as valuable objective measures to capture the heterogeneity in lifestyle factors linked to cognitive decline. We demonstrated that MRS scores are potentially useful for identifying individuals who are more likely to experience cognitive decline.

## RESULTS

### Study datasets

The Health Brain Initiative (HBI) is a longitudinal study of older adults in South Florida, aimed at advancing dementia prevention and promoting brain health.^37^ Our analysis included a total of 88 blood-based DNA methylation samples generated from 88 participants (60 females, 28 males) enrolled in HBI (Table 1). Among the participants, 59 (67.0%) were cognitively normal (CN) and 29 (33.0%) were diagnosed with Mild Cognitive Impairment (MCI). The mean age of the participants at the time of sample collection was 67.95 ± 10.32 years, about half (46.6%) of them smoked, and about a third (29.5%) had at least one *APOE* ε4 allele. Subjects in this dataset are highly educated, with an average of 16.39 years of education.

For external validation, we used DNAm data generated by the longitudinal Alzheimer’s Disease Neuroimaging Initiative (ADNI) study, which included 538 subjects (213 CN, 325 MCI) with available DNAm data (Table 1).^38^ These individuals had a mean age of 74.54 ± 7.51 years at the time of blood draw, and about half (n = 291, 54.09%) were male. Slightly less than half of them were smokers (41.1%) or had at least one *APOE* ε4 allele (37.7%). The subjects in this dataset were also highly educated, with an average of 16.24 years of education.

### Blood DNAm differences at individual CpGs and DMRs are significantly associated with the Resilience Index

**Figure 1:**
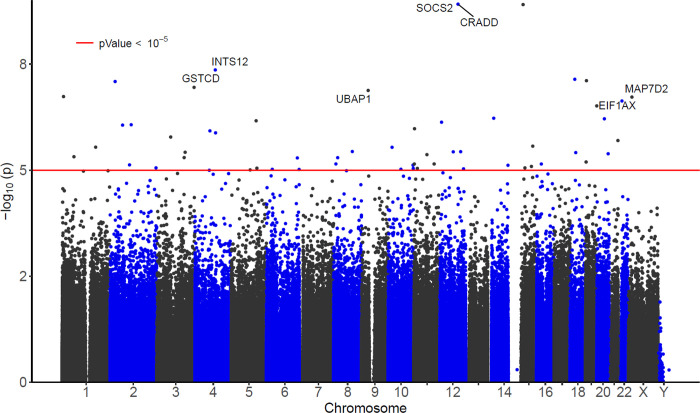
Manhattan plot of significant DNA methylation (DNAm) differences associated with the Resilience Index (RI). The X-axis indicates chromosome number. The Y-axis shows −log_10_(*P-*value) of the association between DNAm M-values and RI, adjusting for covariates (age, sex, diagnosis, cell type compositions, *APOE ε4* allele count). The genes with promoter regions associated with the top 10 most significant CpGs are highlighted.

After adjusting for covariate variables (age, sex, diagnosis, *APOE ε4* allele count, and immune cell type compositions) and correcting genomic inflation (Methods), our robust linear model identified 19 CpGs significantly associated with the Resilience Index at FDR < 0.05 (Figure 1, Table 2, Supplementary Table 1). An additional 37 CpGs were identified at a more relaxed significance threshold of *P* < 1×10^−5^. Among these 56 significant CpGs, about half (27 CpGs, 48.2%) showed hypermethylation associated with increased values of the Resilience Index. About a fifth (12 CpGs, 21.4%) are located in CpG islands, and a quarter (13 CpGs, 23.2%) are located in promoter regions less than 2k bp from the transcription start site (TSS).

Among the most significant CpGs (Table 2), the *UBAP1* gene encodes a protein involved in endosomal trafficking, the process of recycling and degradation of cellular components. Mutations in the *UBAP1* gene have been identified as a cause of the neurodegenerative disorder Juvenile-onset hereditary spastic paraplegias (HSPs). ^39,40^ The *EIF1AX* gene plays a crucial role in translation initiation and is part of the preinitiation complex involved in recruiting ribosomal subunits for protein synthesis. A recent study demonstrated that in the ALS-FUS mutation mouse model, EIF1AX and several other translation initiation factors were downregulated, which led to an impairment in protein translation, mitochondrial dysfunctions, synaptic plasticity impairment, reduced spine density, and cognitive deficits ^41^. Another noteworthy gene is *GSTCD*, which encodes a protein that is part of the glutathione S-transferase (GST) family responsible for detoxifying reactive oxygen species (ROS). Our observed negative association between the promoter region of *GSTCD* and the Resilience index is consistent with previous observations that oxidative stress is a hallmark of various neurodegenerative diseases, and that the glutathione S-transferase is vital for protecting neurons from oxidative damage.^42^

Using *P-*values for individual CpGs as input, comb-p^43^ software identified 24 differentially methylated regions (DMRs), which had Sidak multiple comparison-adjusted *P-*value < 0.05 and a nominal *P*-value < 1×10^−5^, and all the CpGs within the DMR have a consistent direction of change in estimated effect sizes in individual CpG analysis (Supplementary Table 2). The number of CpGs in these DMRs ranged from 3 to 12. Among these DMRs, half showed hypermethylation associated with increased value of the RI (12 DMRs) or are in CpG islands (12 DMRs). A little less than half are located in promoter regions (10 DMRs, 41.7%).

The most significant promoter DMR is in the *APOC2* gene, situated within the APOE-APOC1-APOC4-APOC2 gene cluster on Chromosome 19. *APOC2* encodes an apolipoprotein that serves as a cofactor for lipoprotein lipase (LPL), the enzyme responsible for the breakdown of triglycerides. Dysregulation in apolipoproteins leads to disruption in lipid transport and breakdown, which have been linked to metabolic imbalances and cognitive decline in AD^44^. Among the genes associated with the most significant DMRs (Table 3), *BBS4* encodes a core component of the BBSome protein complex, which is essential for transporting proteins and receptors, including G protein-coupled receptors (GPCRs), to the primary cilium ^45^. Mutations in *BBS4* and other BBS-related genes lead to ciliary dysfunction, resulting in a spectrum of neurodevelopmental impairments (e.g., cognitive deficits, reduced IQ) and metabolic disorders (e.g., obesity, type 2 diabetes) ^46^. *DNAAF5* encodes a cytoplasmic protein that assembles the motors of cilia, which are crucial for fluid movement across tissues such as the brain ventricles, where they help regulate cerebrospinal fluid (CSF) flow. A recent GWAS identified variants in the *DNAAF5* gene that are associated with increased levels of phosphorylated tau (p-tau), a biomarker for AD ^47^. Finally, the *HMGB3* gene encodes a protein from the high-mobility group (HMG) family. The HMGB proteins can function as cytokines in the inflammatory response, which influences neuroinflammation, a key factor known to exacerbate AD pathology ^48^. Together, these findings highlighted the epigenetic regulation of genes involved in endosomal trafficking, protein synthesis, oxidative stress, lipid metabolism, cilia function, and immune responses, all of which may play critical roles in promoting resilience against cognitive decline.

### Pathway analysis revealed DNA methylation differences associated with the Resilience Index are enriched in biological pathways involved in lipid metabolism, energy regulation, and cardiovascular health

To further understand the biological processes underlying the RI, we next performed pathway analysis using the methylGSA software.^49^ At a 5% false discovery rate (FDR), we identified 11 KEGG pathways and 10 Reactome pathways significantly enriched with RI-associated DNAm (Table 4, Supplementary Table 3). Importantly, our analysis highlighted the central role of lipid metabolism and energy regulation in maintaining brain health, the significant pathways included *Glycerophospholipid metabolism*, *Plasma lipoprotein clearance*, *Plasma lipoprotein assembly, remodeling, and clearance*, *Regulation of insulin secretion*, and *NR1H2 and NR1H3 mediated signaling*. Impairments in lipid metabolism and insulin resistance are well known to be associated with cognitive decline, especially in AD. ^50,51^ In addition, our results underscored the connection between cardiovascular health and cognitive function. Several pathways, including *Hypertrophic cardiomyopathy (HCM), Dilated cardiomyopathy, and Cardiac conduction*, emphasize the importance of heart function and blood flow in supporting brain health and preventing cognitive decline. ^52^

Consistent with results from individual CpGs and DMRs, our pathway analysis also identified several additional key mechanisms contributing to cognitive resilience, including proper protein synthesis (Aminoacyl-tRNA Biosynthesis), the maintenance of synaptic plasticity (*Axon guidance, CREB1 phosphorylation through NMDA receptor-mediated activation of RAS signaling*), functional cilia, which ensure neurons respond correctly to external signals such as neurotransmitters (*Cilium assembly, Anchoring of the basal body to the plasma membrane*), preventing the accumulation of damaged proteins (*Ubiquitin-mediated proteolysis*), and maintaining a healthy immune system regulation (*NR1H2 and NR1H3 mediated signaling*)*.* Finally, several KEGG pathways, including *Alzheimer’s disease, Huntington’s disease,* and *Glioma*, were directly associated with cell death and dysfunction in the brain.

### Correlation of significant DNAm differences with expression of nearby genes in blood samples

To evaluate the functional role of the significant DMRs and CpGs, we overlapped our significant DNAm differences with previously established DNAm to RNA expression associations (i.e., eQTM), which were computed from matched blood DNA methylation and gene expression data in a large dataset of more than 4000 subjects from the Framingham study.^53^ Among the 56 significant individual CpGs and those within the 24 DMRs, we found 29 CpGs and 8 CpGs were significantly correlated with target gene expression in *cis* (i.e., within 500k bp of the CpG) or *trans*, respectively (Supplementary Tables 4–5). In particular, the target genes with *cis* associations included *BTBD11, TUBGCP5, HLA-DPB1, HLA-DPA1, CLPTM1,* and *RUFY1*.

The target gene most strongly associated with *cis-*DNAm differences is *HLA-DPB1*, a member of the Human Leukocyte Antigen (HLA) gene family. HLA genes play a vital role in brain health by influencing the body’s ability to eliminate foreign antigens, which is essential for preventing chronic inflammation and autoimmunity - key factors linked to neurodegenerative diseases. Recent genome-wide association studies (GWAS) have identified a number of genetic variants in HLA genes that are associated with age-related brain disorders, such as AD. ^54–56^ Among other genes associated with DNAm in *cis*, *TUBGCP5* encodes a protein involved in microtubule organization. Microtubules are critical for the structural integrity of neurons and the efficient transport of molecules within nerve cells. Defects in microtubules can impair axonal transport, synaptic function, and neuronal stability, and have been linked to various neurodegenerative diseases, including AD. ^57^ The *CLPTM1* gene encodes a transmembrane protein involved in regulating the trafficking and function of inhibitory synaptic receptors, such as GABA-A receptors, which play a key role in maintaining the balance of excitatory and inhibitory signaling in the brain. ^58^ Previous studies have implicated *CLPTM1* with various neurological conditions, including Alzheimer’s disease. ^59,60^ Finally, the *RUFY1* gene encodes a protein that plays a crucial role in intracellular trafficking and cytoskeleton regulation. It is involved in several essential processes, such as endocytosis, autophagy, and endosomal trafficking, which are critical for transporting molecules within cells and maintaining cellular homeostasis. Genetic variants in the *RUFY1* gene, along with other genes involved in endo-lysosomal transport, have been associated with early-onset AD in a recent study. ^61^ These findings revealed that the RI-associated DNAm may influence the expression of nearby target genes, to affect the risk of AD and other brain disorders.

### Correlation and overlap with genetic risk loci

We identified methylation quantitative trait loci (mQTLs) by comparing our RI-associated CpGs with blood mQTLs in the GoDMC database.^62^ Among the 56 significant individual CpGs (Supplementary Table 1) and the 134 CpGs located in significant DMRs (Supplementary Table 2), 93 CpGs had 97988 mQTLs in *cis* and 19 CpGs had 4702 mQTLs in *trans* in the blood (Supplementary Table 6).

Next, we evaluated if the mQTLs overlapped with genetic risk loci implicated in dementia, by comparing them with the genetic variants nominated in a recent ADRD meta-analysis.^63^ We found that while no mQTLs overlapped with the genome-wide significant loci, 549 SNPs overlapped with genetic variants reaching a suggestive genome-wide significance threshold at *P* < 10^−5^ (Supplementary Table 7).

Given the observed overlap between the mQTLs and ADRD genetic risk loci, we next sought to determine whether the association signals at these loci (variant to CpG methylation levels and variant to clinical ADRD status) were due to a single shared causal variant or distinct causal variants close to each other. To this end, we performed a co-localization analysis using the method described by Giambartolomei et al. (2014)^64^. The results of this co-localization analysis strongly suggested^65^ (i.e. PP3+PP4 > 0.90, PP4 > 0.8, and PP4/PP3 > 5) that one genomic region, located in the *HLA-DPA1 and HLA-DPB1*genes, included a single causal variant common to both phenotypes (i.e. ADRD status and CpG methylation levels). (Supplementary Table 8).

### Correlation of RI-associated DNAm in blood and brain samples

To better understand the role of the RI-associated DNAm in brain health, we sought to prioritize methylation differences with a consistent direction of change in both blood and brain. To this end, we analyzed the London dataset ^66^, which includes matched DNAm samples measured on postmortem brain and pre-mortem blood samples of 69 subjects ^67^ (Supplementary Table 9). For each CpG, we computed Spearman rank correlations between DNA methylation levels in the brain and blood. We performed an unadjusted correlation analysis based on methylation beta values ($r_{beta}$), as well as an adjusted correlation analysis based on methylation residuals ($r_{resid}$), obtained after removing effects of estimated neuron proportions for brain samples (or estimated blood cell-type proportions), batch, age at death (for brain samples) or at blood draw (for blood samples), and sex.

Among the 189 significant individual CpGs and CpGs mapped within the DMRs, 30 CpGs showed significant brain-to-blood associations in both the adjusted and unadjusted analyses (${FDR}_{beta}<0.05$, ${FDR}_{resid}<0.05$) (Supplementary Table 10). Notably, all these CpGs were located within DMRs, with the majority (28 out of 30 CpGs, 93.3%) showing significant positive correlations between brain and blood. Intriguingly, among these 30 CpGs with significant brain-to-blood correlations, two-thirds of them (20 CpGs, 66.6%), mapped to the *TUBGCP5*, *APOC2*, *RUFY1*, and *HLA-DPA1* genes, also showed a significant DNAm-to-mRNA association in *cis* in blood samples (Supplementary Table 4). Intriguingly, 7 CpGs, located on the *HLA-DPB1*, *HLA-DPA1*, and *HCG24* genes, also showed significant DNAm-to-RNA associations in brain samples (Supplementary Tables 9,11). Together, these findings highlight DNAm at these 7 CpGs, which are predominantly located within DMRs, show consistent changes in both brain and blood and influence downstream gene expression. These characteristics make them excellent candidates for biomarkers of brain health.

### The majority of Resilience Index-associated DNAm are associated with CSF AD biomarkers, AD brain neuropathology, clinical AD status, or aging in independent studies

To validate our findings, we compared the RI-associated CpGs and DMRs identified in our study with those from previous research using our recently developed MIAMI-AD database (https://miami-ad.org/).^68^ Among the 56 significant CpGs measured on the EPIC v2 platform, 44 were also available on the earlier EPIC v1 or 450k platforms. Our comparison showed that the majority of these CpGs (42 CpGs, 95.5%) overlapped with significant findings from previous studies, with changes occurring in the expected directions (Supplementary Table 12). In previous studies, these CpGs were significantly associated with CSF AD biomarkers (6 CpGs), AD brain neuropathology (4 CpGs), clinical AD status (7 CpGs), and aging (41 CpGs). The corroborated CpGs are located in promoter regions of the genes *MTA1, NPAS2, ZIC1, SOCS2, SEMA7A, RAPGEF6, RPUSD2*, SSH3, and intergenic regions.

Similarly, among the 133 CpGs located in DMRs, 120 were also available on the earlier platforms. The majority of these CpGs (113 CpGs, 94.2%) overlapped with significant findings from previous studies, with a consistent direction of change (Supplementary Table 13). In previous studies, these CpGs were significantly associated with CSF AD biomarkers (14 CpGs), AD brain neuropathology (20 CpGs), clinical AD status (33 CpGs), and aging (103 CpGs). The corroborated CpGs are located in genes *TUBGCP5, APOC2, EME2, NME3, ITIH1, BBS4, HLA-DPA1, NEDD1, DNAAF5, MRPS34, HMGB3*, *LAX1* and intergenic regions.

### Out-of-sample validation demonstrated that Methylation-based Resilience Scores predicted AD progression in an external cohort

To assess the feasibility of using RI-associated DNAm to predict AD progression, we developed an MRS score. To this end, we fitted an elastic net model with the Resilience Index as the outcome and significant CpGs, both individual CpGs and CpGs located in DMRs, as predictors to the HBI dataset. This model selected 54 CpGs with non-zero weights, ranging from −2.77 to 3.61, with positive weights assigned to 22 CpGs and negative weights to 32 CpGs. Notably, the directions of these weights were concordant with the estimated effect sizes from robust linear models for all 54 CpGs (Supplementary Table 14). Moreover, these weights were significantly associated with the estimated effect sizes from the robust linear models (Spearman correlation = 0.747, *P-*value = 9.16 ×10^−11^).

We conducted an out-of-sample validation of the MRS using an external dataset from the ADNI study, which included 538 subjects with available DNAm data and follow-up visit information (Table 1). We analyzed the earliest available (baseline) DNAm sample from each subject. These subjects were followed for an average of 5.39 ± 2.94 years, with follow-up durations ranging from 0.44 to 11.71 years. By their last visit, 64 (30.0%) CN subjects had progressed to MCI or AD, and 131 (40.3%) MCI subjects had progressed to AD.

**Figure 2:**
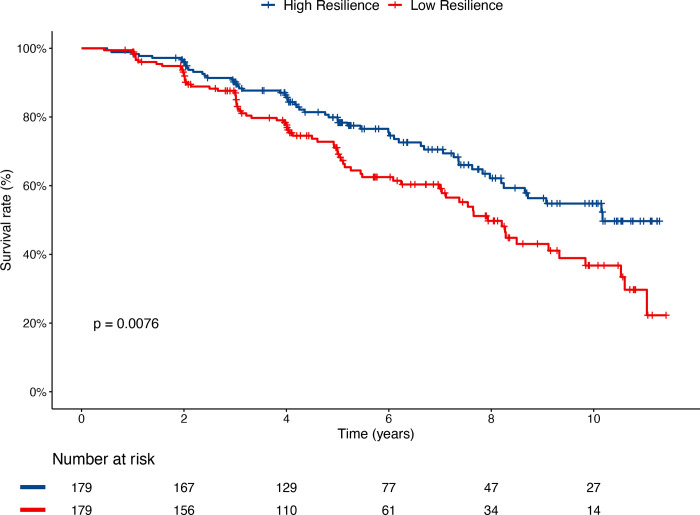
Kaplan-Meier curve for Alzheimer’s disease progression (CN to MCI/AD, or MCI to AD) among subjects with the highest and lowest tertiles of baseline MRS score in the ADNI cohort. While survival probability decreases in both groups, the high resilience group consistently shows a higher survival probability, suggesting a protective effect of higher resilience against AD progression. **Abbreviations** CN, cognitively normal; MCI, mild cognitive impairment; AD, Alzheimer’s disease; MRS, Methylation-based Resilience Score

For each of these 538 subjects, we computed MRS scores using DNAm data from their baseline visit and evaluated their association with disease progression (i.e., CN to MCI/AD, MCI to AD) using the Cox regression model. After adjusting for age, sex, *APOE ε4* status, years of education, baseline diagnosis, and baseline MMSE score, the MRS was significantly associated with progression to the next disease stage (estimate = − 0.035, *P-*value = 4.06 × 10^−3^) (Table 5). The Kaplan-Meier curve (Figure 2) demonstrates that subjects in the highest and lowest MRS tertiles had significantly different survival probabilities over time (*P-*value = 0.0076). In particular, while survival probability decreases in both groups, the high resilience group consistently shows a higher survival probability, indicating a protective effect of higher resilience against AD progression.

## DISCUSSION

Despite its significance, cognitive resilience remains a largely abstract concept with limited biological understanding. In this study, we conducted a comprehensive analysis to identify DNAm differences associated with the Resilience Index, a validated measure of six lifestyle factors relevant to cognitive health. ^28^ To ensure the robustness of our findings, we employed several strategies. First, we used robust linear regression models which were designed to mitigate the impact of outlier samples. Second, we addressed genomic inflation by applying the bacon method, ^69^ specifically developed for epigenome-wide association studies (EWAS). Finally, we adjusted for potential confounding variables in our test of DNAm-to-RI associations, which included age, sex, diagnosis, number of APOE ε4 alleles, and the first two principal components of immune cell type proportions.

After correcting for multiple comparisons, our analysis identified 19 individual CpGs and 24 DMRs significantly associated with the RI in the HBI cohort. The most significant DNAm changes were found in genes involved in endosomal trafficking and lipid metabolism (*UBAP1, APOC2, CLPTM1, RUFY1*), cilia function (*BBS4, DNAAF5*), protein synthesis (*EIF1AX*), oxidative stress (*GSTCD*), and neuroinflammation (*HMGB3, HLA-DPB1*). Notably, two CpGs located in the promoter of the *APOC2* gene (cg09555818 and cg13119609) were among the top 20 CpGs reaching genome-wide significance in a recent EWAS study of over 3,500 subjects from the Generation Scotland study that compared *APOE* ε4 versus ε2 carriers ^70^. Consistent with the hypomethylation at the *APOC2* locus observed in carriers of the *APOE* ε4 allele, which increases the risk of AD substantially, we found that promoter hypermethylation at *APOC2* was associated with the Resilience Index. Results from our pathway analysis also underscored the strong connection between cardiovascular and brain health, highlighting significant pathways such as *Hypertrophic cardiomyopathy*, *Dilated cardiomyopathy*, and *Cardiac conduction*. Recent studies have increasingly recognized the heart-brain connection, demonstrating that even subclinical cardiac damage is a significant risk factor for dementia.^52,71–73^ Thus, maintaining heart health is crucial for promoting brain health.

In comparing the significant DNAm differences with findings from previous studies, we found that over 85% of the RI-associated CpGs were also involved in aging, a process known to induce widespread DNAm changes (Supplementary Tables 12–13). The association of these CpGs with both aging and the RI suggests they may serve as biomarkers of healthy aging, reflecting not only the passage of time but also the preservation of brain function and resilience against neurodegeneration. Arenaza-Urquijo and Vemuri (2018) discussed two mechanisms by which lifestyle factors could prevent or delay AD: resistance, or reducing AD neuropathology, and resilience, or maintaining cognitive performance despite pathology. We found that a number of the significant RI-associated CpGs (10 out of 44 CpGs, 22.73%) had been previously reported to be significantly associated with cerebrospinal fluid (CSF) AD biomarkers or brain AD neuropathology. This suggests that the DNAm at these CpGs might influence AD by reducing neuropathological burden. Future studies that comprehensively investigate the mechanisms by which RI-associated DNAm influences AD are needed.

By identifying DNAm associated with the RI, our study provided an alternative approach to discovering future targets and treatment strategies for AD, complementary to the traditional method of identifying risk variants directly linked to the disease. For example, efforts are already underway to develop *APOC2* mimetic peptides, aimed at restoring normal lipid metabolism by enhancing lipoprotein lipase (LPL) activity, which holds promise not only for treating cardiovascular diseases but also for addressing lipid-related brain health issues.^74^ Another example is the *TUBGCP5* gene involved in microtubule organization. A recent study in amyloidogenic mouse models demonstrated that microtubule-stabilizing agents reduced tau and Aβ levels, promoting both neuronal and cognitive protection.^75^

A significant challenge in advancing precision medicine for AD is the heterogeneity in the disease risk among individuals, and a key factor contributing to this variability is cognitive resilience.^5^ Previous studies have shown that DNAm is associated with several lifestyle factors thought to influence cognitive resilience, including smoking, diet, exercise, obesity, alcohol consumption, and HDL cholesterol.^76–79^ Using external samples from the ADNI study, we demonstrated that our MRS was significantly associated with disease progression (i.e., from CN to MCI/AD, or MCI to AD), after accounting for covariates age, sex, *APOE ε4* status, education, baseline diagnosis, and baseline MMSE scores. This provides a proof-of-concept that DNAm biomarkers may help identify individuals at greater risk of cognitive decline.

This study has several limitations. First, given the relatively small sample size of our discovery cohort (n = 88), we performed our analyses using all available samples, which included samples from both CN and MCI subjects. To test the association between DNAm and the RI, we employed a robust linear model that adjusted for diagnosis, which ensures that our findings are applicable for both diagnostic groups. To further validate these results, we conducted sensitivity analyses by fitting the robust linear models separately to CN (n = 59) and MCI (n = 29) subjects. We found the Spearman correlation between the estimated effect sizes in the all-sample analysis and the CN-only analysis was 0.74 (*P-*value < 2.2 × 10^−16^), with all estimated effect sizes showing the same direction of change (Supplementary Table 15). Similarly, the Spearman correlation between the estimated effect sizes in the all-sample analysis and the MCI-only analysis was 0.55 (*P-*value < 2.2 × 10^−16^), with all but two CpGs (cg13462576 and cg01655777) showing the same direction of change. Therefore, the estimated effect sizes of the DNAm-to-RI associations in the CN-only and MCI-only analyses were largely consistent with those from the all-samples analysis. Future studies with larger sample sizes of CN and MCI subjects will be crucial for identifying DNAm changes uniquely associated with the RI in each population.

Similarly, although our dataset included a small number of samples from Black (n = 11), Asian (n = 10), and Hispanic (n = 9) subjects, we did not have enough sample size to analyze each race and ethnicity separately. Moreover, the subjects in our dataset are highly educated with 16 years of education on average. The HBI is an ongoing longitudinal study with a racially and ethnically diverse cohort, including many participants with lower educational levels. Future large-scale, multi-ethnic DNA methylation studies incorporating diverse backgrounds are needed to identify DNAm biomarkers of cognitive resilience in minority and underrepresented populations.

In summary, our study provided new insights into the epigenetics underlying cognitive resilience and highlights the potential of methylation biomarkers as objective measures of resilience. We identified a number of CpGs and DMRs associated with the RI, many of which are involved in lipid metabolism, protein synthesis, oxidative stress, and neuroinflammation - key processes implicated in AD and cognitive aging. These significant RI-associated DNA methylation differences could point to novel targets and treatment strategies for AD, complementing traditional approaches that identify disease-associated variants directly. As the neurodegenerative process underlying AD is challenging to halt or reverse, the importance of prevention strategies has increasingly been recognized. Our results suggest that MRS could help capture the heterogeneity in lifestyle factors linked to cognitive decline and offers a promising approach for identifying individuals more likely to maintain cognitive function as they age, and more importantly, those most likely to experience cognitive decline. Future studies with larger, more diverse cohorts are needed to further validate these findings and explore the potential of DNAm biomarkers for personalized medicine in AD.

## METHODS

### Study participants

The participants in this study were recruited from the ongoing longitudinal Healthy Brain Initiative (HBI) study, which conducts comprehensive assessments of cognitive, medical, physical, and brain health in residents of South Florida. DNA methylation was extracted from blood samples provided by a subset of participants during their baseline HBI visit. Our analysis included 88 participants with complete HBI baseline data relevant to the current investigation.

Exclusion criteria included age under 50 years, lack of consent for data or specimen storage, absence of a study partner, or a diagnosis of moderate to severe Alzheimer’s disease or related dementias (Clinical Dementia Rating [CDR] ≥ 2). Additional exclusions were made for participants unable to provide clinical, cognitive, behavioral, or functional data, or those with significant medical conditions that could interfere with neuroimaging or cognitive outcomes, such as metastatic cancer, major psychiatric disorders, unstable chronic diseases, or substance abuse within the past five years. All participants provided informed written consent in accordance with procedures approved by the University of Miami Institutional Review Board. The study protocol and procedures have been described in detail elsewhere. ^37^

The external validation dataset consisted of 538 whole blood DNAm samples from the ADNI study.^38^ This dataset included 213 CN and 325 MCI subjects at their first visit with available DNAm data (baseline). Conversion events were defined as the progression of CN subjects to MCI or AD, and the progression of MCI subjects to AD. Follow-up data were censored at the point of loss to follow-up, death from non-dementia causes, or final follow-up date of the study.

### DNA methylation experiment and pre-processing

To generate HBI cohort DNAm data, genomic DNA (500 ng) was bisulfite-treated to convert unmethylated cytosines to uracil using the EZ-96 DNA Methylation^™^ Kit (Zymo Research). CpG methylation was measured using the Illumina Infinium MethylationEPIC v2.0 Beadchips at the Center for Genomic Technology (CGT), John P. Hussman Institute for Human Genomics (HIHG).

Supplementary Table 16 shows the number of CpGs and samples at each quality control (QC) step. Specifically, QC for CpG probes involved removing probes with a detection *P-*value < 0.01 in all samples using the minfi R package. Additional criteria for probe removal included probes that are cross-reactive^80^, do not start with “cg” in the probe ID, or contain a single nucleotide polymorphism (SNP) with a minor allele frequency (MAF) ≥ 0.01 within the last 5 base pairs of the probe. This step was implemented using the DMRcate R package (with the rmSNPandCH function and parameters dist = 5, mafcut = 0.01).

For quality control of samples, we verified that all samples had high bisulfite conversion efficiency (> 85%). Furthermore, the methylation data-predicted sex (using watermelon R package’s estimateSex function) matched the recorded sex for all samples. We removed two outlier samples identified in principal component analysis (PCA), defined as those falling outside ±3 standard deviations from the mean of PC1 and PC2. Samples from subjects lacking information on the Resilience Index (RI) were also excluded. The final analysis included 88 DNA methylation samples, all of which were processed in a single batch on one methylation plate.

The quality-controlled DNA methylation dataset were then subjected to the QN.BMIQ normalization procedure^81^, implemented using the lumN function in the lumi R package, followed by the BMIQ function in the wateRmelon R package. As recommended ^82^, we did not normalize probes on the X chromosome, but retained them for analyses. Immune cell type proportions (B lymphocytes, natural killer cells, CD4+ T cells, CD8+ T cells, monocytes, neutrophils, and eosinophils) were estimated using the EpiDISH R package. Consistent with previous blood-based DNAm studies ^83–85^, granulocyte proportions were computed as the sum of neutrophils and eosinophils proportions, as both cell types are classified as granular leukocytes.

The DNAm samples from the ADNI study were pre-processed similarly as described above, with adjustments due to the larger sample size of this dataset. Specifically, we selected probes with a detection *P-*value < 0.01 in 90% or more of the samples. Also, we corrected for batch effects from methylation plates using the BEclear R package ^86^.

### Statistical analyses to identify DNA methylation significantly associated with Resilience Index

For each CpG, we fitted a robust linear model with DNAm M-values as the outcome, the RI as the primary independent variable, and relevant covariates. Given our relatively modest sample size (n = 88), we included age, sex, diagnosis, APOE ε4 allele count, and the first two principal components of immune cell type proportions (which accounted for 90.7% of the variance in all estimated cell-type proportions). Although we considered additional factors such as smoking, race, and ethnicity, they were not significantly associated with the RI (*P*_smoking_ = 0.221, *P*_Black_ = 0.488, P_Asian_ = 0.205, P_Hispanic_ = 0.368), and were therefore less likely to confound our analysis.

For region-based meta-analysis, we used the comb-p method ^43^. Briefly, comb-p takes single CpG *P-*values and locations of the CpG sites to scan the genome for regions enriched with a series of adjacent low *P*-values. In our analysis, we used *P*-values from the robust linear model as input for comb-p. We used parameter settings with --seed 0.05 and --dist 750 (a *P*-value of 0.05 is required to start a region and extend the region if another *P*-value was within 750 base pairs), which were shown to have optimal statistical properties in our previous comprehensive assessment of the comb-p software^87^. As comb-p uses the Sidak method to account for multiple comparisons, we selected DMRs with Sidak *P*-values less than 0.05. To help reduce false positives, we imposed two additional criteria in our final selection of DMRs: (1) the DMR also has a nominal *P*-value < 1×10^−5^; (2) all the CpGs within the DMR have a consistent direction of change.

### Inflation assessment and correction

Genomic inflation factors (lambda values) were estimated using both the conventional approach ^88^ and the *bacon* method ^69^, which was specifically designed for EWAS. The lambda value (λ) from the conventional approach was 1.03, and the lambda value from the bacon method (λ.bacon) was 0.99. Although inflation correction was not strictly necessary, we applied it to maintain consistency with our analysis pipeline. After inflation correction using the bacon R package, the lambda values remained unchanged, with λ=1.03 and λ.bacon=1.00.

### Functional annotation and pathway analysis

Significant methylation at individual CpGs and differentially methylated regions (DMRs) was annotated using gene annotations from GENCODE v41, as provided in Supplementary Table 7 of Noguera-Castells et al. (2023) ^89^, and the Genomic Regions Enrichment of Annotations Tool (GREAT) ^90^ software, which associates genomic regions with target genes.

To identify biological pathways enriched with RI-associated DNAm differences, we used the methylRRA function from the methylGSA R package ^49^, which takes single CpG *P*-values as input. Briefly, methylGSA first computes a gene-wise ρ value by aggregating *P*-values from multiple CpGs mapped to each gene. It then adjusts for the different numbers of CpGs per gene using Bonferroni correction. Finally, a Gene Set Enrichment Analysis ^91^ is performed in pre-ranked analysis mode to identify pathways enriched with significant CpGs. We analyzed pathways from the KEGG and REACTOME databases, focusing on those with 2 to 200 genes. To avoid gene sets where the enrichment signal is driven by only one or two genes, we additionally required the significant gene sets include at least three genes in the “core enrichment” subset. Pathways with an FDR less than 0.05 were considered statistically significant.

### Integrative analyses with gene expression, genetic variants, and brain-to-blood correlations

To evaluate the effect of DNAm on the expression of nearby genes in blood samples, we overlapped our RI-associated DNAm, including both significant individual CpGs and those located within DMRs, with eQTm analysis results in Supplementary Tables 2 and 3 of Yao et al. (2021)^53^.

In brain sample analysis of DNAm-to-RNA associations, we used matched RNA-seq gene expression data and methylation data from 511 subjects in the ROSMAP study ^92^. We computed the correlations between significant CpGs and the expressions of genes located within ± 250 kb from the start or end of the CpG. To minimize false positives due to noise from lowly expressed genes, we included only genes with FPKM > 0.5 in at least 10 unaffected samples and 10 affected samples. To reduce potential confounding effects, we adjusted for cell-type proportions, age at death, sex, and batch effects in methylation *M* values (and separately for log_2_ transformed gene expression values) by fitting linear models and extracting residuals. Finally, we tested for associations between CpG methylation residuals and gene expression residuals, adjusting for Braak stage.

To assess the correlation of RI-associated DNAm in blood and brain samples, we used the London dataset, which consisted of 69 samples with matched PFC and blood samples ^67^. We assessed the association of brain and blood methylation levels at RI-associated CpGs using both an unadjusted correlation analysis with methylation beta values ($r_{beta}$), and an adjusted correlation analysis using methylation residuals ($r_{resid}$), in which we removed the effect of estimated neuron proportions in brain samples (or estimated immune cell-type proportions in blood samples), array, age at death (for brain samples) or age at blood draw (for blood samples), and sex.

For correlation and overlap with genetic susceptibility loci, we searched for mQTLs in the blood using the GoDMC database (http://mqtldb.godmc.org.uk/downloads). ^62^ To select significant blood mQTLs in GoDMC, we used the same criteria as the original study, ^62^ that is, considering a cis *P-*value smaller than 10^−8^ and a trans *P-*value smaller than 10^−14^ as significant. The genome-wide summary statistics for genetic variants associated with dementia described in Bellenguez et al. (2022) ^63^ were obtained from the European Bioinformatics Institute GWAS Catalog (https://www.ebi.ac.uk/gwas/) under accession no. GCST90027158. Colocalization analysis was performed using the coloc R package.

### Validation using independent datasets

To compare our results with previous findings, we searched RI-associated CpGs (both significant individual CpGs and those located in DMRs) using the CpG Query tool in the MIAMI-AD database^93^ (https://miami-ad.org/). For the input on phenotype, we selected “AD Biomarker”, “AD Neuropathology”, “Aging”, “Dementia Clinical Diagnosis”, and “Mild Cognitive Impairment”.

In addition, we evaluated the predictive ability of RI-associated DNAm in relation to the disease progression of MCI or AD. To this end, we developed MRS scores, calculated using significant individual CpGs and those located within DMRs. Specifically, we first estimated the coefficients of CpGs using an elastic net model trained on the HBI dataset, with the RI as response variable. The model parameters, λ and α, were optimized via five-fold cross validation based on the mean squared error (MSE). After tuning, the final model was fitted with parameters log(λ)=15.66 and α=0.1, yielding an error within one standard error of the minimum MSE. The final model selected 54 CpGs with non-zero weights (Supplementary Table 14).

To assess disease progression, we performed out-of-sample validation on the ADNI data using the MRS by summing the methylation M-values for the CpGs with non-zero weights and adding the intercept obtained from the final model. We then conducted Cox proportional hazards regression analyses on the ADNI dataset to evaluate the association between MRS and disease progression. The analyses were performed using the coxph function in the survival R package. Disease progression was defined as the conversion from CN to MCI or AD, and from MCI to AD. The model was adjusted for multiple covariates: Surv (Conversion event, follow-up time) ~ MRS + age + sex + APOE ε4 status + years of education + baseline diagnosis + baseline MMSE score. Furthermore, we separated the samples into three groups based on tertiles of their baseline MRS scores: low resilience (MRS < 192.44), medium resilience (192.44 ≤ MRS < 197.51) and high resilience (MRS ≥ 197.51). To assess the different conversion probabilities over time in the low and high resilience groups, we performed a log-rank test to determine whether the conversion distributions of these two groups were significantly different. Additionally, we visualized the progression patterns using the Kaplan-Meier curves, which provided a clear representation of how the probability of conversion to MCI or AD varied over time in each group. This allowed us to observe whether individuals in the low resilience group had a higher or faster rate of disease progression compared to those in the high resilience group.

## Figures and Tables

**Table 1:** Characteristics of the participants included in this study from the Health Brain Initiative and Alzheimer’s Disease Neuroimaging Initiative studies.

		HBI (Discovery)			ADNI (Validation)	
Characteristics	All	CN	MCI	All	CN	MCI

Sample size, n	88	59	29	538	213	325
Age, av. (sd)	67.95 (10.32)	66.25(9.36)	71.41 (11.43)	73.54 (7.52)	76.72 (6.56)	73.12 (7.76)
Sex, n (%)						
Female	60 (68)	43 (73)	17 (59)	247 (46)	106 (50)	141 (43)
Male	28 (32)	16 (27)	12 (41)	291 (54)	107 (50)	184 (57)
Education in years, av. (sd)	16.39 (2.71)	16.68 (2.48)	15.79 (3.71)	16.24 (2.65)	16.36 (2.66)	16.17 (2.65)
Smoking history, n (%)						
Yes	41 (47)	30 (51)	11 (39)	221 (41)	92 (43)	129 (40)
No	45 (51)	28 (47)	17 (61)	317 (59)	121 (57)	196 (60)
APOE4, n (%)	26 (30)	18 (31)	8 (24)	203 (38)	52 (24)	151 (47)

**Table 2:** Top 10 most significant CpGs associated with the Resilience Index (RI) in the analysis of blood samples from the HBI cohort. The robust linear model included methylation M value as the outcome, resilience index (RI) in z-scores as the main independent variable, and covariate variables age, sex, diagnosis (MCI, CN), APOE4 allele count, and the first two PCs of the estimated immune cell-type proportions. Estimates describe changes in DNA methylation M values associated with a one standard deviation increase in RI after adjusting for covariate variables. A positive (or negative) value of the Estimate indicates hypermethylation (or hypomethylation) of the CpG associated with increased values in RI. In GREAT annotations, gene promoter regions are highlighted in red text, and the numbers in parentheses indicate distance from the TSS.

CpG	Estimate	StdErr	pValue	fdr	chr	position	GREAT_annotation

cg22125370	−0.175	0.029	1.23E-09	5.58E-04	chr12	93,572,709	CRADD (−105008);SOCS2 (+1028)
cg01684389	−0.214	0.035	1.26E-09	5.58E-04	chr15	31,065,241	MTMR10 (−73637);TRPM1 (+96032)
cg03215315	−0.184	0.034	4.36E-08	1.21E-02	chr4	105,708,826	GSTCD (+8);INTS12 (+282)
cg23351331	0.235	0.044	7.24E-08	1.21E-02	chr18	24,160,113	CABYR (+21018);OSBPL1A (+237746)
cg24419436	−0.257	0.048	7.81E-08	1.21E-02	chr19	4,446,034	UBXN6 (+11788);CHAF1A (+43372)
cg02959866	0.214	0.040	8.17E-08	1.21E-02	chr2	26,142,102	GAREM2 (−30989);RAB10 (+107992)
cg05170851	0.154	0.029	1.12E-07	1.42E-02	chr3	193,897,344	HES1 (−238801);OPA1 (+304200)
cg13582261	0.118	0.022	1.33E-07	1.47E-02	chr9	34,180,004	UBAP1 (+999)
cg16559708	0.300	0.058	1.85E-07	1.69E-02	chr1	7,070,435	VAMP3 (−700834);CAMTA1 (+285111)
cg00459767	−0.173	0.033	1.90E-07	1.69E-02	chrX	20,139,847	MAP7D2 (−22930); EIF1AX (+1997)

**Table 3:** Top 10 most significant differentially methylated regions (DMRs) associated with Resilience Index, identified by the comb-p software. Direction indicates hypermethylation (+) or hypomethylation (-) at each CpG that are associated with increased values of Resilience Index. Annotations were based on the GREAT software, where the numbers in parentheses indicate distance from the TSS. Gene promoter regions associated with DMRs are highlighted in red text.

DMR	width	n_probes	P-value	Sidak-P	direction	GREAT_annotation

chr2:240623007–240623931	925	6	2.71E-12	2.60E-09	++++++	GPR35 (+4526);AQP12B (+59431)
chr19:2543604–2544103	500	5	6.94E-12	1.23E-08	-----	GADD45B (+67726);GNG7 (+158856)
chr5:179559290–179559830	541	12	1.10E-09	1.81E-06	------------	RUFY1 (+9002);HNRNPH1 (+65109)
chr19:44945836–44946045	210	6	8.87E-09	3.77E-05	++++++	APOC2 (−1263)
chr15:72685411–72685700	290	5	1.35E-08	4.15E-05	+++++	BBS4 (−632);HIGD2B (+594)
chr7:726222–726483	262	4	6.57E-08	2.23E-04	----	DNAAF5 (−349)
chr8:57143317–57143617	301	4	9.41E-08	2.78E-04	----	FAM110B (−851087);IMPAD1 (−149623)
chr12:109131254–109131376	123	5	4.16E-08	3.02E-04	+++++	ACACB (+14720);FOXN4 (+177905)
chr5:191432–191692	261	5	2.30E-07	7.85E-04	-----	SDHA (−26679);PLEKHG4B (+51304)
chrX:150983099–150983351	253	11	3.00E-07	1.06E-03	+++++++++++	HMGB3 (−61)

**Table 4:** A total of 11 KEGG pathways and 10 Reactome pathways were significantly enriched with Resilience Index-associated CpGs at 5% FDR, using the methylGSA (PMID: 30346483) software.

Pathway	Size	pValue	FDR

***KEGG pathways***			
HYPERTROPHIC_CARDIOMYOPATHY_HCM	78	5.01E-06	9.07E-04
DILATED_CARDIOMYOPATHY	85	1.04E-05	9.37E-04
UBIQUITIN_MEDIATED_PROTEOLYSIS	128	5.81E-05	3.51E-03
AMINOACYL_TRNA_BIOSYNTHESIS	38	1.67E-04	7.54E-03
GLYCEROPHOSPHOLIPID_METABOLISM	74	2.12E-04	7.67E-03
GLIOMA	63	3.08E-04	8.42E-03
AXON_GUIDANCE	127	3.26E-04	8.42E-03
CIRCADIAN_RHYTHM_MAMMAL	13	1.46E-03	3.28E-02
ALZHEIMERS_DISEASE	145	1.63E-03	3.28E-02
ETHER_LIPID_METABOLISM	32	2.35E-03	4.25E-02
HUNTINGTONS_DISEASE	161	2.94E-03	4.84E-02
***Reactome pathways***			
RECRUITMENT_OF_MITOTIC_CENTROSOME_PROTEINS_AND_COMPLEXES	78	1.10E-06	1.68E-03
NR1H2_AND_NR1H3_MEDIATED_SIGNALING	44	1.19E-04	3.03E-02
AURKA_ACTIVATION_BY_TPX2	70	6.37E-05	3.03E-02
ANCHORING_OF_THE_BASAL_BODY_TO_THE_PLASMA_MEMBRANE	95	1.19E-04	3.03E-02
CARDIAC_CONDUCTION	119	7.16E-05	3.03E-02
PLASMA_LIPOPROTEIN_CLEARANCE	33	3.08E-04	4.29E-02
PLASMA_LIPOPROTEIN_ASSEMBLY_REMODELING_AND_CLEARANCE	71	2.39E-04	4.29E-02
REGULATION_OF_INSULIN_SECRETION	73	2.85E-04	4.29E-02
CILIUM_ASSEMBLY	193	2.89E-04	4.29E-02
CREB1_PHOSPHORYLATION_THROUGH_NMDA_RECEPTOR_MEDIATED_ACTIVATION_OF_RAS_SIGNALING	28	4.08E-04	4.78E-02

**Table 5:** Results from Cox regression model evaluating the association between Methylation-based Resilience Score (MRS) and disease progression (CN to MCI/AD, MCI to AD) in 538 subjects, adjusted for age, sex, APOE ε4 status, baseline diagnosis, MMSE, and education. Significant association was observed for MRS (estimate = −0.035, *P*-value = 0.00406), indicating higher MRS reduces risk of progression in AD.

Characteristic	Coefficient	HR (95% CI)	*P*-value

**MRS**	−0.035	0.965 (0.943,0.989)	4.06E-03
**Age, years**	0.065	1.067 (1.045,1.089)	6.36E-10
**Sex Male**	0.081	1.085 (0.801,1.470)	6.00E-01
***APOE* ε4 allele**			
None		1 [Reference]	
>=1	0.775	2.171 (1.627,2.897)	1.36E-07
**Baseline diagnosis**			
CN		1 [Reference]	
MCI	0.503	1.654 (1.190,2.300)	2.76E-03
**MMSE**	−0.134	0.832 (0.761,0.910)	6.17E-05
**Education, years**	−0.017	0.984 (0.928,1.043)	5.80E-01

**Abbreviations:** MRS, Methylation-based Resilience Score; HR, hazard ratio; CI, confidence interval; CN, cognitively normal; MCI, Mild Cognitive Impairment; MMSE, Mini-Mental State Examination

## Data Availability

The ADNI dataset can be accessed from http://adni.loni.usc.edu, the ROSMAP dataset can be accessed from AD Knowledge Portal (accession: syn3157275), and the HBI dataset can be accessed from GEO database (accession: GSE281429).
